# Understanding Cellular, Molecular, and Functional Specificity, Heterogeneity, and Diversity of the Endocannabinoid System

**DOI:** 10.3390/cells13121049

**Published:** 2024-06-17

**Authors:** Jun Aoki, Masako Isokawa

**Affiliations:** 1 Graduate School of Frontier Biosciences, Osaka University, Osaka 565-0871, Japan; junaoki@fbs.osaka-u.ac.jp; 2 Center for Biosystems Dynamics Research, Laboratory for Cell Signaling Dynamics, RIKEN, Kobe 650-0047, Japan; 3 Forefront Research Center, Graduate School of Science, Osaka University, Osaka 560-0043, Japan

The endocannabinoid system (ECS) is a widely recognized lipid messenger system involved in many aspects of our health and diseases. The system consists of cannabinoid receptors, their endogenous ligands, and the enzymes that mediate the synthesis and metabolic processes of the ligand, endocannabinoid. Research progress was reviewed recently in (i) the pharmacology of ECS, (ii) the roles of ECS in development and synaptic function, (iii) cannabinoid signaling in pathological conditions, and (iv) cell-type specific and localization-dependent operation of cannabinoid receptors [1,2]. Of particular interest may be the conceptual framework where biomedical consequences influenced by ECS are not necessarily monotheistic; instead, there exists cellular, molecular, and functional specificity, heterogeneity, and diversity in (endo)cannabinoid action.

This Special Issue discusses the multiplexity of the endocannabinoid signaling mechanism and function and how data are visualized for presentation. It is suggested that data mining strategies and atlas-based data profiling often help identify the involvement of critical molecules throughout our lives [3,4]. For example, methodological orientation provided typical standards for non-parametric dimensionality reduction, data visualization, and cluster analysis that ranged from traditional Principal Component Analysis (PCA) to the currently popular Uniform Manifold Approximation and Projection (UMAP) [5,6,7]. Although some researchers may feel these approaches are still young and relatively underexplored in endocannabinoid research, fruitful applications have emerged in investigating ligands, receptors, and related enzymes.

The first example concerns the role of ECS in the transition of retinal Müller glia (MG) into proliferating progenitor-like cells in health and diseases [8,9]. In this work, single-cell RNA sequencing was used, and eCB-related gene expression was reported in the formation of Müller glia-derived progenitor cells (MGPCs) after NMDA-induced damage. UMAP analysis served as a tool for dimensionality reduction for a fuller understanding of the contribution of the ECS and fatty acid signaling in the reactivity and dedifferentiation of Müller glia, as well as the proliferation of microglia and MGPCs.

The second example is regarding the involvement of cannabinoid receptor type-1 (CB1R/Cnr1) and type-2 (CB2R/Cnr2) in nonalcoholic fatty liver disease (NAFLD) [10] and melanoma [11]. Although the deletion of Cnr1 (CB1R KO mice) was hypothesized to prevent the development of NAFLD, scRNA-seq and UMAP analysis did not support the hypothesis and pointed out that Cnr1−/− mice failed to protect the liver from fibrosis. In addition, UMAP analysis portrayed a positive correlation between the upregulation of intra-tumoral CB2R gene expression and improved overall survival in melanoma.

Anandamide synthesis was found to be increased in the partial hepatectomy (PHX) model [12]. Global Transcriptomic Analysis illuminated the upregulation of cell-cycle proteins and their transcriptional regulator and provided molecular and genetic evidence to show that the conjugation of arachidonic acid and ethanolamine by fattyacid amide hydrolase was involved in the pathophysiology of PHX.

Finally, it may be worth mentioning that, in pharmaceutical sciences, dimensionality reduction and cluster analysis such as t-distributed stochastic neighbor embedding (t-SNE) and UMAP have been frequently used on a large-scale sample of cannabis sativa chemotypes for modeling cannabinoids [13]. Here, a dataset of 17,600 commercial cultivars was screened for unknown gene regulation and pharmacokinetics of dozens of cannabinoids. The concentration of tetrahydrocannabinol (THC) scattered against the concentration of cannabidiol (CBD) was plotted to segregate high- and low-CBD and THC cultivars. These approaches helped reveal complex interactions in cannabinoid biosynthesis and advanced the phenotypical classification of cannabis cultivars.

In sum, we introduced examples of some methodological tools for data generation and analysis. scRNA-seq provides a way of comprehensively defining gene expression and identifying molecular trajectories by connecting the transcriptomes. However, the reconstruction of molecular lineages from gene expression cascades to cell-type specific markers and regulators is still a major challenge. We also introduced some of the techniques in data science that reduce the dimensionality of raw data and visualize the outcome in a pictorial format, i.e., t-SNE, UMAP, heatmap, dendrogram, and violin plots. They are routinely applied in a broad range of fields, including life sciences, where increasing sizes of datasets are handled. While these techniques have been used liberally in combination with transcriptomics and proteomics, less usage was seen in the detection of molecules that cannot be labeled with antibodies and/or genetic manipulation. Further applications are encouraged with all-inclusive measurement technologies in cells and organisms such as imaging mass spectrometry, as it can directly detect and visualize identified and unidentified lipids and metabolites that often play key roles in the eCB system.

We hope you will find the collection of papers in this Special Issue interesting and helpful for the advancement of single-cell and tissue-based investigations in the eCB system. We also hope that this Special Issue helps you to expand the methodological choices for mining, summarizing, and presenting new ideas and perspectives for future eCB research.
